# Cost-effectiveness of surgical interventions for the management of osteoarthritis: a systematic review of the literature

**DOI:** 10.1186/s12891-017-1540-2

**Published:** 2017-05-10

**Authors:** Hanin Kamaruzaman, Philip Kinghorn, Raymond Oppong

**Affiliations:** 1 Malaysian Health Technology Assessment Section, Ministry of Health, Putrajaya, Malaysia; 2 Health Economics Unit, Institute of Applied Health Research, University of Birmingham, Birmingham, UK

**Keywords:** Osteoarthritis, Cost-effectiveness, Costs, Review

## Abstract

**Background:**

The primary purpose of this study is to assess the existing evidence on the cost-effectiveness of surgical interventions for the management of knee and hip osteoarthritis by systematically reviewing published economic evaluation studies.

**Methods:**

A systematic review was conducted for the period 2004 to 2016. Electronic databases were searched to identify both trial and model based economic evaluation studies that evaluated surgical interventions for knee and hip osteoarthritis.

**Results:**

A total of 23 studies met the inclusion criteria and an assessment of these studies showed that total knee arthroplasty (TKA), and total hip arthroplasty (THA) showed evidence of cost-effectiveness and improvement in quality of life of the patients when compared to non-operative and non-surgical procedures. On the other hand, even though delaying TKA and THA may lead to some cost savings in the short-run, the results from the study showed that this was not a cost-effective option.

**Conclusions:**

TKA and THA are cost-effective and should be recommended for the management of patients with end stage/severe knee and hip OA. However, there needs to be additional studies to assess the cost-effectiveness of other surgical interventions in order for definite conclusions to be reached.

**Electronic supplementary material:**

The online version of this article (doi:10.1186/s12891-017-1540-2) contains supplementary material, which is available to authorized users.

## Background

Osteoarthritis (OA) is the most common form of joint disease, and results from a progressive degenerative change in the joint structure. OA is associated with any joint in the body but the most commonly affected are the hip and the knee [1]. It has been estimated that about 251 million people suffer from knee OA worldwide [2]. The prevalence of OA increases with age, and with the constant rise in the global ageing population [3, 4], the economic burden of the disease is also likely to rise.

Osteoarthritis places a strain on scarce resources. For example, in a recent study, the total annual direct cost of osteoarthritis in the US was estimated to be double that of similar patients who did not have osteoarthritis [5]. In the UK, the total health care cost of osteoarthritis is estimated at over £1 billion (2010 prices) [6]. Based upon national survey data, Kortlarz et al. estimate the increased insurer expenditure for women in the US with osteoarthritis to be $4,833 [7]. For men with osteoarthritis, the additional insurer cost was estimated as $4,036 [7]. There is therefore a need for cost-effective approaches for the management of OA.

Treatment and management of OA involves a multidisciplinary approach and various management options include patient education and self-management, non-pharmacological treatments and pharmacological treatments. In the recently updated clinical guidelines for management of OA, issued by the National Institute for Health and Care Excellence (NICE), comprehensive and integrated care which involves healthcare professionals, patients and carers are among the key recommendations intended to ensure the maximum benefit for patients [8]. Apart from these pharmacological and non-pharmacological interventions, there are also surgical interventions which are more expensive and are normally limited to those patients who do not respond to other forms of treatment. Due to the increase in the number of older people, there has been an increase in the demand for surgical interventions such as joint arthroplasty which has caused a rise in the costs associated with OA [9–11].

Systematic reviews have assessed the cost-effectiveness of pharmacological and non-pharmacological treatments for OA [12–14]. However to the best of our knowledge, a systematic review of economic evaluations assessing surgical interventions for the management of OA has never previously been published or reported. This study is therefore aimed at reviewing published economic evaluations aimed at assessing and summarizing evidence on the cost-effectiveness of surgical interventions for the management of OA. The focus is on knee and hip OA since they are the most common forms of OA.

## Methods

The search and identification of papers followed a two-stage process. The initial search included economic evaluations associated with *any* clinical trials or cohort studies that assessed surgical, non-pharmacological and pharmacological interventions for *all types* of OA. This was necessary to meet the needs of the wider research project. The search strategy outlined below reflects this initial, broad scope.

### Search strategy

Electronic databases (Ovid MEDLINE(R) In-Process & Other Non-Indexed Citations and Ovid MEDLINE(R), Ovid EMBASE Classic and EMBASE, EBM Reviews-Cochrane Database of Systematic Review, EBM Reviews-NHS Economic Evaluation Database, PubMed, INAHTA database and HTA websites) were searched for economic evaluations associated with any clinical trials or cohort-based trials that assessed all types of treatment modalities (pharmacological, non-pharmacological, surgical) for all types of OA.

Additional literature which was considered as potentially relevant was identified from the bibliographies of the retrieved articles and search engines such as Google Scholar. Any studies with limited access were retrieved by emailing the author(s) and no language restrictions were imposed. The searches were conducted between 2004 and 2016. This timeframe was chosen for pragmatic reasons and in order to capture the most recent studies. The detailed search terms that were used, has been outlined in Additional file 1.

### Selection of studies

All titles and abstracts of retrieved articles were screened and any duplicates were excluded from the initial list. Each article was then checked for suitability in terms of their potential in assessing the cost-effectiveness and economic analysis of various treatment modalities for management of OA. Full text articles of these selected titles were retrieved and reviewed for further selection in accordance to pre-specified inclusion and exclusion criteria. The selection of articles was carried out independently by two reviewers [HK] and [RO]. Results from each reviewer were compared and any differences were resolved through consultation among reviewers.

It was from this broad pool of papers that the following, more specific, inclusion and exclusion criteria were applied, in order to identify studies falling within the scope of this paper.

### Inclusion criteria

The inclusion criteria for this systematic review were divided into 5 main components as follows:

i. Population: Patients with knee or hip osteoarthritis
ii. Intervention: Surgical interventions (total knee arthroplasty, total hip arthroplasty etc).
iii. Comparators: Any comparators (no interventions, usual care, and other surgical modalities)
iv. Outcomes: Any outcomes for economic evaluations (cost effectiveness, incremental cost-effectiveness ratio or ICER)
v. Study design: Cost consequence analysis, cost-benefit analysis, cost-effectiveness analysis, cost-utility analysis both trial-based studies and model-based economic analysis

### Exclusion criteria

The exclusion criteria for this review were as follows:

i. Study designs that do not consider knee or hip OA or study designs that combine OA patients with other types of arthritis (rheumatoid arthritis, lupus arthritis, ankylosing spondylitis)
ii. Partial or non-economic evaluation studies
iii. Ongoing studies and protocol
iv. Systematic reviews, narrative review, commentaries and letters
v. Duplicated publications

### Data extraction and methods of analysis and synthesis

The following data was extracted from the selected studies: Study characteristics such as type of economic analysis, population, interventions, comparators, perspective, time horizon, and types of modelling used, effectiveness and cost measures, main results, ICER, base-case outcomes and sensitivity analyses being used. A summary of the studies relating to surgical interventions for the treatment of hip and knee OA is presented in Table 1.

**Table 1:** Economic evaluation summary evidence table

No.	Author & year	Country	Intervention (s)	Comparator (s)	Study design	Economic study type	Perspective	Study population	Measure of effectiveness	Cost types / Currency / Price year	Discount rates
1.	Bedair et al, [18]	USA	Total knee arthroplasty (TKA)	Non-operative treatment	Markov model	CBA	Societal	Severe unilateral knee OA	Cost saving approximately 3.5 years after surgery	Direct and indirect costs / US dollar / 2012	3% for costs and effects
2.	Bozic et al, [29]	USA	Metal-on-metal hip resurfacing arthroplasty (MoM-HRA)	Total hip arthroplasty (THA)	Markov model	CUA	Healthcare system	Patients with advanced hip OA	QALYs	Direct costs / US dollar / price year not clearly stated (2008/2009)	5% for costs and effects
3.	Di Tanna et al, [39]	Italy	Cementless fixation technique for THA	Hybrid fixation technique for THA	Markov model	CEA	Healthcare provider	Patients with hip OA undergoing THA	"revision-free" life year	Only prosthesis, surgical and revision costs / Euro / price year not stated	3.5% for costs and effects
4.	Heintzbergen et al, [37]	Canada	MoM-HRA	Conventional THA	Markov model	CUA	Healthcare system	Patients with hip OA undergoing hip arthroplasty	QALYs	Direct costs / Canadian dollar / 2011	3% for costs and effects
5.	Higashi et al, [35]	Australia	Total replacement of hips and knees	‘Doing nothing’ (non-surgical therapies without joint replacements)	Discrete event simulation model	CUA	Healthcare system	68,908 with hip OA and 100,657 with knee OA	DALYs (disability-adjusted life-years)	Direct costs / Australian dollar / 2003	3% for costs and effects
6.	Koskinen et al, [21]	Finland	Unicondylar arthroplasty (UKA)	TKA	Register-based analysis	CEA	Not mentioned	Knee OA patients undergoing either UKA or TKA or both	"revision-free" life year	Only prosthesis costs (3 different UKA implants) / Euro / 2003	Nil
7.	Li et al, [22]	Germany	KineSpring Knee Implant System	No treatment, conventional treatments or other surgical interventions for knee OA	Not reported	CUA	Not mentioned	Mild-to-moderate knee OA patients (not eligible for TKA /UKA)	QALYs	Direct and indirect costs (no details given) / Euro / 2012	Nil
8.	Losina et al, [36]	USA	TKA done in low, medium & high volume hospitals	No TKA performed	Markov model	CUA	Societal	Patients with end-stage knee OA for TKA	QALYs	Direct and indirect costs / US dollar / 2006	3% for costs and effects
9.	Mota, [38]	Italy	Early primary THA	1. Non-surgical then primary THA 2. Non-surgical with NSAIDs	Markov model	CUA	Healthcare provider	Patients with hip OA undergoing hip arthroplasty	QALYs	Direct costs / Euro / 2010	3% for costs and effects
10.	Pennington et al, [41]	UK / England	Cementless and hybrid prosthesis for THA	Cemented prosthesis for THA	Markov model	CUA	Healthcare system	OA patients aged 55 to 84 undergoing THA	QALYs	Direct costs only / British pound sterling / 2010-2011	3.5% for costs and effects
11.	Räsänen et al, [28]	Finland	1. Primary THA 2. Secondary / revision THA 3. Primary TKA	Non-operative intervention	Cohort-based study	CUA	Healthcare provider	Cohort of patients from 30 medical entities in Finland	QALYs	Direct hospital costs only / Euro / price year not stated	5% for effects (QALY) only
12.	Ruiz et al, [31]	USA	TKA	Non-operative treatment	Markov model	CUA	Societal	Adults aged 40 years old and older undergoing TKA for knee OA in the USA in 2009	QALYs	Direct and indirect costs / US dollar / 2009	3% for costs and effects
13.	SooHoo et al, [25]	USA	UKA	TKA	Decision tree	CUA	Societal	Unicompart-mental knee OA patients	QALYs	Direct costs only / US dollar / 1998	3% for costs and effects
14.	Suter et al, [26]	USA	“innovative” TKA implants (highly crossed-linked polyethylene or other innovative biomaterials)	“standard” implants (an ultra-high molecular weight all polyethylene tibial component)	Markov analysis based on Osteoarthri-tis Policy (OAPol) Model	CUA	Societal	Adults with symptomatic end stage knee OA needing TKA	QALYs	Direct costs only / US dollar / 2010	3% for costs and effects
15.	Waimann et al, [32]	USA	TKA	Hypothetical nonsurgery strategy	Cohort-based study	CEA	Societal	212 patients with knee OA who under-went TKR.	Improvement in WOMAC score	Direct and indirect costs / US dollar / 2007	Nil
16.	Xie et al, [27]	Canada & Singapore	TKA	UKA	Cohort-based study (hospital in Singapore)	CUA	Societal	431 TKR patients and 102 UKA patients	QALYs	Direct costs only / US dollar / 2008	Nil
17.	Konopka et al, [33]	USA	HTO and UKA	TKA	Markov model	CUA	Societal	Patients with knee OA requiring surgery	QALYs	Direct and indirect costs / US dollar / 2012	3% for costs and effects
18.	Marsh et al, [19]	Canada	Arthroscopic surgery in addition to non-operative treatments	Non-operative treatments alone (optimised physical and medical therapy)	Alongside RCT	CEA and CUA	Healthcare and societal	Patients with knee OA	WOMAC Index QALYs	Direct and indirect costs / Canadian dollar / 2014	Nil
19.	Mather et al, [20]	USA	Primary TKA without delay	Delayed TKA (with and without non-operative bridge treatment)	Markov model	CUA	Healthcare and societal	End-stage knee OA	QALYs	Direct and indirect costs / US dollar / 2009	3%
20.	Peersman et al, [34]	Belgium	UKA	TKA	Markov model	CUA	Healthcare	Patients with unicondylar knee OA	QALYs	Direct costs / Euro / 2014	1.5% and 3%
21.	Pennington et al, [23]	UK	Different brands within types of hips prosthesis (cemented, cementless and hybrid)	-	Markov model	CUA	Not mentioned	Patients with hip OA requiring primary THA	QALYs	Direct costs / British pounds sterling / 2010/2011	3.5%
22.	Pulikottil-Jacob et al, [40]	UK	Combinations of components in hip prosthesis for THA, including the type of fixation and bearing surfaces	-	Markov model	CUA	NHS and Personal Social Services	Patients undergoing THA for hip OA	QALYs	Direct costs / British pound sterling / 2012	3.5%
23.	Stan et al, [24]	Romania	Unilateral TKA and TKA following HTO	Rehabilitation care	Alongside clinical trial	CUA	Not mentioned	Patients with knee OA	QALYs	Direct costs / Euro / year not mentioned	3% (outcome only)

### Assessment of the quality of studies

Assessment of the quality of included studies was performed by using the Consolidated Health Economic Evaluation Reporting Standards or CHEERS [15] (Additional file 1) and Philips criteria checklist [16] (Additional file 1) for model based studies. All included studies were also quantitatively assessed using the Quality of Health Economic Studies (QHES) instrument (Additional file 1) which was designed to evaluate the appropriateness of the methodology, the validity and transparency of the study results and the comprehensiveness of reporting the study itself [17].

## Results

### Search results

**Fig. 1:**
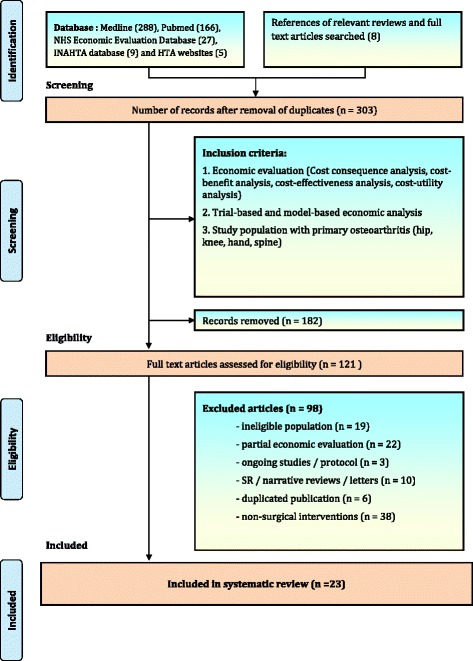
Flow diagram of the study selection process

The initial (broad) search yielded 303 potentially relevant articles and after reviewing the abstracts and applying the inclusion criteria, 121 studies were initially included for full text review. After applying exclusion criteria set out above, a total of 98 articles were excluded, mainly because they were duplicates (6 studies), were not concerned with assessing surgical interventions (38 studies), not limited to knee and hip OA patients (19 studies), not full economic evaluations (22 studies), protocols (3 studies) and systematic reviews (10 studies). A total of 23 studies were included in the final sample for this paper (Fig. 1).

### Summary of selected studies

All studies included in this review were published between 2004 and 2016 and were conducted across nine different countries (USA, Italy, Canada, Australia, Finland, UK, Singapore, Belgium and Romania). The types of economic evaluations conducted were mainly cost-utility analysis (78%) and cost-effectiveness analysis (13%) with one study conducting a cost-benefit analysis [18] and another conducting both a cost-utility analysis and a cost-effectiveness analysis [19]. Sixteen studies were model based, whilst six were trial based (Table 1). Most studies adopted either a healthcare (nine studies) or a societal perspective (eight studies). Two studies however adopted both a healthcare and a societal perspective [19, 20]. Of the remaining studies, four did not specifically mention the perspective that was adopted [21–24].

Three studies only incorporated direct medical costs in their analysis even though they stated that they adopted a societal perspective [25–27]. Most studies described the comparators that were used; one study stated that the comparator of choice was a non-operative strategy [28] but did not give any specific details about the non-operative strategy used. For studies that used the QALY as an outcome measure, various health-related quality of life (HR-QoL) instruments such as the SF-36, EQ-5D, SF-6D, 15-Dimensional Utility Index (15D), Health Utility Index (HUI) and Health Assessment Questionnaire (HAQ) were used. Two studies [25, 29], however, did not specifically mention the tools that were used to derive the QALY estimates. Apart from the generic HR-QoL instruments, some studies utilised disease-specific health state instruments. For instance, Räsänen et al [28] used Harris Hip Score (HHS) and Knee Society Score (KSS) as complementary disease-specific tools for the 15D generic instrument used in the study. Other disease-specific tools used were Oxford Hip Score (OHS), Oxford Knee Score (OKS) and Western Ontario and McMaster Universities Osteoarthritis Index (WOMAC). The most widely used tool by healthcare providers in assessing OA patients is WOMAC Osteoarthritis Index which consists of 24 items used to evaluate pain, stiffness and physical functions of OA patients in daily living [30].

### Cost-effectiveness of interventions for the management of knee and hip OA

For surgical modalities for knee OA, the most common intervention was total knee arthroplasty whilst the most common surgical modality for hip OA was total hip arthroplasty.

### Cost-effectiveness of Total Knee Arthroplasty (TKA)

#### TKA versus non-surgical/non operative strategies

Four studies compared TKA to non-operative/non-surgical strategies [18, 28, 31, 32] and all studies concluded that TKA is a cost-effective intervention. Of these, three studies adopted a societal perspective [18, 31, 32] whilst the fourth study [28] adopted a healthcare provider perspective. The aim of the first study [18] was to assess the cost-effectiveness of TKA in the younger working population and used a Markov model to assess the cost-effectiveness of TKA over a 30 year period in a hypothetical 50 year old patient with severe OA. The second study also used a Markov model to estimate the lifetime cost-effectiveness of TKA in patients with end stage OA of the knee [31]. The remaining two studies [28, 32] assessed the short term cost-effectiveness of TKA using trial-based and cohort studies and did not limit the population included in their respective studies by age or stage of the disease. A summary of results is presented in Table 2.

**Table 2:** Summary of main results

No.	Author & Year	Intervention(s) evaluated	Key result(s)	Conclusion	QHES scores^a^
1.	Higashi et al, [35]	Total replacement of hip and knee	Both hip and knee replacements were cost-effective compared to 'doing nothing' at the pre-defined threshold level of AUD 50,000 per DALY. • THR : AUD 7100 to 15000 per DALY with different time cost & cost offset • TKR : AUD 15000 to 26000 per DALY with different time cost & cost offset	Both hip and knee replacements are cost-effective interventions to improve the quality of life of people with OA.	70
2.	Bedair et al, [18]	Total knee arthroplasty (TKA)	Treatment with TKA has a higher initial cost, but the cost benefit in favor of TKA approximately 3.5 years after surgery (a difference of US$69,800 over the same time period when treated with non-operative strategies	The total economic cost to society for treatment of severe knee OA in a relatively young working person is markedly lower with TKA than it is with non-operative treatment.	74
3.	Losina et al, [36]	Total knee arthroplasty (TKA) performed in low, medium and high volume hospitals	- Base-case ICER : US$18300 / QALY - If willingness to pay (WTP) to improve QOL were set at US$50 000 per QALY, TKA had a 93% chance of being the preferred choice (ie, TKA had the highest net benefit) compared with no TKA. - Low-risk patients : 96% chance that TKA would be preferred to no TKA if WTP US$50 000 per QALY - High-risk patients : 83% chance that TKA would be preferred to no TKA	- TKA appears to be cost-effective in the US Medicare-aged population, as currently practiced across all risk groups. - Policy decisions should be made on the basis of available local options for TKA. - However, when a high-volume hospital is available, TKAs performed in a high-volume hospital confer even greater value per dollar spent than TKAs performed in low-volume centres.	73
4.	Ruiz et al, [31]	Total knee arthroplasty (TKA)	- Relative to nonsurgical treatment, the mean lifetime net societal savings per patient resulting from TKA was US$18,930 - Each TKA increased lifetime direct costs by a mean of US$20,635, while the societal savings in lower indirect costs from improved functional status averaged US$39,565. - Considering only direct costs, the average ICER was US$5656 per QALY gained for TKA in the entire cohort and US$12,410 for those of 80 years old and older.	Overall, TKA was cost-effective across all age groups, assuming a willingness-to-pay threshold of US$50,000 per QALY gained taken from societal perspective.	71
5.	Waimann et al, [32]	Total knee arthroplasty (TKA)	- The ICERs for WOMAC improvement at 6 months were as follows: 1) US$33,345 to achieve an MCID 2) US$25,255 per each WOMAC20 improvement, 3) US$35,274 per each WOMAC50 improvement 4) US$56,908 per each WOMAC70 improvement - TKA would be a cost-effective intervention if the WTP amount for the minimum clinically significant absolute or relative improvement were US$50,000.	Although there was no established WTP value for WOMAC change, TKA appeared to be a cost-effective intervention for end-stage knee OA at both low and high levels of improvement in the patients’ pain and function.	60
6.	Xie et al, [27]	Total knee arthroplasty (TKA)	- ICUR was US$65,245/QALY from the societal perspective. - The probability of TKA being a cost-effective strategy is less than 0.4 from the societal or patients’ perspective if the WTP is US$50,000/QALY. - In contrast, the probability that TKR is a cost effective strategy is 0.7 from the government’s perspective if the WTP is only US$10,000/QALY	TKA gained more QALYs at higher costs compared to UKA. However, a long-term prospective study is necessary to determine the cost-effectiveness of TKR and UKA.	62
7.	Koskinen et al,[21]	Unicondylar knee arthroplasty (UKA)	- The mean cost of one revision from UKA to TKA was €8,660 including implant, hospital stay, operation, and other direct costs. Thus, the costs saved by lower implant prices and shorter hospital stay for UKA as compared to TKA would not cover the costs of the extra revisions.	At a nationwide level, UKA had significantly poorer long-term survival than TKA. UKA did not even have a theoretical cost benefit over TKA in the study. Based on the results, widespread use of UKA in the treatment of unicompartmental OA of the knee cannot be recommended.	33
8.	SooHoo et al, [25]	Unicondylar knee arthroplasty (UKA)	- In reference case, UKA has only small gain of QALY (0.02) and minimal increment in costs, from US$18,995 to US$19,000 compared to TKA - Reference case ICER : US$277 per QALY gained. - In lower durability / survival of UKA in terms of function, UKA becomes less effective and more costly. - If durability / survival of TKA is longer (range 15 to 20 years), TKR becomes more cost effective. - If TKR durability is 20 years, the ICER for UKA would be $45,958 per QALY gained when UKA is assumed to be functional up to 17 years (^a^below threshold)	This analysis demonstrates the potential for UKA to be a cost-effective alternative to TKA, depending on the cost as well as the durability and function of a UKA.	59
9.	Li et al, 2013 [22]	KineSpring Knee Implant System - intermediate treatment between conservative care and joint-altering surgery targeting the treatment gap in knee OA patients.	- Assuming the durability of 10 years, the cost-utility ratio of each intervention compared to no treatment : • KineSpring : €3,402 ± 4,168/QALY, • Surgical interventions : €4,899 ± 1,094/QALY • Conservative treatments : €9,996 ± 13,612/QALY	The KineSpring Knee Implant System for knee OA is a cost-effective strategy over other surgical and conservative treatments for patients in Germany.	44
10.	Suter et al, [26]	“innovative” TKA implants	- Innovative implants offered ≥50% decrease in long-term TKA failure at ≤50% increased cost offered ICERs < US$100,000 regardless of age or baseline comorbidity. - Innovative implant provided a 20% decrease in long-term failure at 50% increased cost provided ICERs < US$150,000 per QALY gained only among healthy 50–59-year-olds. - Increasing short-term failure, consistent with recent device failures, reduced cost-effectiveness in all groups.	Innovative implants must decrease actual TKA failure, not just radiographic wear, by 50–55% or more over standard implants to be broadly cost-effective.	65
11.	Mota, [38]	Early primary THA	- Early THA has cost-effectiveness ratios of €4100 or below in all cases. - Among 80-year-olds, early THA is (extended) dominant = ICER of €20,406. - Delayed THA is not cost-effective at any threshold for base-case scenario. - At age 65 years, the ICER for THA over delayed THA was €987 in men and €466 in women.	In summary, results suggest that THA is a cost-effective treatment option, and in general should be offered without delay to functionally independent patients with severe OA.	82
12.	Räsänen et al, [28]	1. Primary THA 2. Secondary / revision THA 3. Primary TKA	- The cost per QALY gained (ICUR) was lowest in the primary THA group , followed by primary TKA & revision THA. • Primary THA : €6710 per QALY gained • Primary TKA : €13,995 per QALY gained • Revision THA : €52,274 per QALY gained	Hip and knee replacement both improve HRQoL. The cost per QALY gained from knee replacement is twice that gained from hip replacement.	49
13.	Bozic et al, [29]	Metal-on-metal hip resurfacing arthroplasty (MoM-HRA)	- Lowest ICER [most cost-effective] : men age 55 to 64 (US$28,614/QALY gain) - Three groups with ICER below threshold [below US$50,000/QALY gained) : • men age 55 to 64 (as above) • women younger than 55 (US$47,468/QALY gained) • men younger than 55 (US$48,882/ QALY gained)	MoM-HRA could be clinically advantageous and cost-effective in younger men and women. Further research on the comparative effectiveness of MoM-HRA versus THA should include assessments of the quality of life and resource use in addition to the clinical outcomes associated with both procedures.	82
14.	Heintzbergen et al, [37]	Metal-on-metal hip resurfacing arthroplasty (MoM-HRA)	- Base-case : MoM HRA dominates with -CAD $583 and mean difference QALY 0.079. - With WTP at CAD$50,000/QALY gain, probability HRA is cost-effective are:- • base case : 58% • male 60 years : 9% • male 40 years : 92% - most cost-effective - The results and uncertainty in base-case analyses suggest that in terms of cost-effectiveness, there is little difference between MoM HRA and THA. - In terms of gender, MoM HRA was preferable in men and THA in women - Age wise, MoM HRA was preferable in younger patients and THA in older patients	On average, MoM-HRA was preferred to THA for younger and male patients, but THA is still a reasonable option if the patient or clinician prefers given the small absolute differences between the options and the confidence ellipses around the cost-effectiveness estimates.	81
15.	Di Tanna et al, [39]	Cementless fixation technique for THA	- Base-case ICER : €2402 per "revision-free" life year - Cementless strategy dominant for patients up to 42-y-old (i.e., less costly and more beneficial compared with the hybrid solution) - 43-yr-old onwards, it still remains more effective but with an additional cost : the resulting ICERs showed a direct proportionality to increasing age - From CEAC: • the cementless intervention as a strategy with a high probability (0.88) of being cost effective at 70 y from values of WTP above €2400 • In case of a 75-y-old patient with WTP of €9000, a cementless approach results cost effective with a probability of 0.89.	Following a deterministic sensitivity analysis, hybrid and cementless fixation showed a dominance profile for patients older than 83 y and younger than 43 y, whereas for all ages in between, there is a progressive increase in the ICER of cementless prostheses.	62
16.	Pennington et al, [41]	Cementless and hybrid prosthesis for THA	- The ICER for a hybrid prosthesis compared with a cemented prosthesis was about £2100 per QALY for men and £2500 for women. - For men aged 60 or 80 and for women aged 60, hybrid prostheses gave the highest expected net benefit and had the highest probability of being the most cost effective prosthesis. - For women aged 80, cemented prostheses were most cost effective. - Hybrid prostheses remained likely to be the most cost effective option for men and women aged 70.	- Cemented prostheses are the cheapest option, but hybrid prostheses lead to greater gains in mean post-operative quality of life and are the most cost effective alternative for most patients. - Cementless prostheses do not improve health outcomes sufficiently to justify their higher costs.	82
17.	Konopka et al, [33]	High tibial osteotomy (HTO) and unicompartmental knee arthroplasty (UKA)	- Base case QALYs : 14.62 (HTO), 14.63 (UKA) and 14.64 (TKA). - Discounted total direct medical costs : $20,436 (HTO), $24,637 (UKA) and $24,761 (TKA) - ICER for TKA: $231,900/ QALY - ICER for UKA: $420,100/ QALY - PSA: At a WTP threshold of $50,000 per QALY, HTO was cost-effective 57% of the time; TKA 24%; and UKA 19%. - At a WTP threshold of $100,000 per QALY, high HTO 43% of time, TKA 31%; and UKA 26%.	In 50 to 60-year-old patients with medial unicompartmental knee OA, HTO is an attractive option compared with UKA and TKA The cost-effectiveness of HTO and of UKA depends on rates of conversion to TKA and the clinical outcomes of the conversions.	78
18.	Marsh et al, [19]	Arthroscopic surgery (partial resection and debridement of degenerative meniscal tears and/or articular cartilage) in addition to non-operative treatments	- The ICER was $140.94 (societal), or $120.83 (payer) per one-point improvement on the 2400 point WOMAC total score, translating to $28,188 (societal) and $24,166 (payer) for a clinically important improvement. - The ICUR was equal to − $110,569 (societal) or − $94,792.50 (payer) per QALY gained, where the negative value indicates paying more for a worse outcome. - Uncertainty estimates suggest that even if WTP $400 000 to achieve a clinically important improvement in WOMAC score, or ≥ $50 000 for an additional QALY, there is <20% probability that the addition of arthroscopy is cost-effective compared with nonoperative therapies only.	Arthroscopic debridement of degenerative articular cartilage and resection of degenerative meniscal tears in addition to nonoperative treatments for knee OA is not an economically attractive treatment option compared with non-operative treatment only, regardless of willingness-to-pay value.	74
19.	Mather et al, [20]	Primary TKA without delay	- In the base case, a 2-year wait-time both with and without a non-operative treatment bridge resulted in a lower number of average QALYs gained (11.57 (no bridge) and 11.95 (bridge) vs. 12.14 (no delay). - The ICER comparing wait-time with no bridge to TKA without delay was $2,901/QALY. - When comparing TKA without delay to waiting with non-operative bridge, TKA without delay produced greater utility at a lower cost to society.	TKA without delay is the preferred cost-effective treatment strategy when compared to a waiting for TKA without non-operative bridge. TKA without delay is cost saving when a non-operative bridge is used during the waiting period. As it is unlikely that patients waiting for TKA would not receive non-operative treatment, TKA without delay may be an overall cost-saving health care delivery strategy.	76
20.	Peersman et al, [34]	UKA	- UKA was associated with cost reduction compared with primary TKA of –€2,807 and a utility gain of 0.04 QALYs. UKA was therefore considered superior to TKA. - Analysis determined that the model is sensitive to clinical effectiveness, and that a marginal reduction in the clinical performance of UKA would lead to TKA being the more cost-effective solution. - The acceptability curve shows that the probability that the ICER falls below the threshold of: €10,000 (77.1%) , €25,000 (65.1%) and €50,000 (60.5%).	UKA yields clear advantages in terms of costs and marginal advantages in terms of health effects, in comparison with TKA.	72
21.	Pennington et al, [23]	Different brands within types of hips prosthesis (cemented, cementless and hybrid)	For women with OA aged 70 years, the Exeter V40 Elite Plus Ogee had the lowest risk of revision (5.9% revision risk, 9.0 QALYs) and the CPT Trilogy had the highest QALYs (10.9% revision risk, 9.3 QALYs). - Compared with the Corail Pinnacle (the most commonly used brand), the CPT Trilogy is most cost effective, with an incremental net monetary benefit of £876. - Differences in cost effectiveness between the hybrid CPT Trilogy and Exeter V40 Trident and the cementless Corail Pinnacle and Taperloc Exceed were small.	The hybrid CPT-Trilogy was the most cost effective brand but differences with the hybrid Exeter V40-Trident and the cementless Corail-Pinnacle and Taperloc-Exceed were small. Our study shows the importance of linking PROMs with data on rates of revision after THA but given the extended period of recovery after a THA, collecting further PROMs and QoL beyond the first six months after THA is an important next step which would strengthen future economic evaluations of brands of hip prostheses.	57
22.	Pulikottil-Jacob et al, [40]	- Metal head (cemented stem) on cemented polyethy-lene cup, CeMoP - Metal head (cement-less stem) on cement-less hydroxyapetite coated metal cup (polyethylene liner), CeLMoP - Ceramic head (cementless stem) on cementless hydroxyl-apetite coated metal cup (ceramic liner), CeLCoC - Hybrid metal head (cemented stem) on cementless hydroxyl-apetite coated metal cup (polyethylene liner), HyMoP - Ceramic head (cemented stem) on cemented polyethy-lene cup, CeCoP	- base-case analysis : At a WTP £20,000 per QALY, a cemented prosthesis with metal-onpolyethylene or ceramic-on-polyethylene bearings had the greatest probability of being cost-effective for all groups of age and gender over a lifetime. - The differences in QALYs between categories were extremely small and differences in mean costs were borderline, between only £2550 and £3000 over a lifetime for all comparisons, irrespective of age or gender. - There are large uncertainties, particularly regarding the costs of prostheses and the estimates of lifetime QOL.	On the basis of such small differences and such considerable uncertainties, it is difficult to make a comparison between the cost-effectiveness of different types of prosthesis. Until better data dealing with costs and outcomes become available, it is difficult to justify the recommendation of one type of device over another on considerations of cost effectiveness alone. The choice of prosthesis should be determined by rates of revision, local costs and the preferences of both the surgeon and the patient	62
23.	Stan et al, [24]	- Unilateral TKA (G2) - TKA following HTO (G3)	- No statistically significant differences was found between G2 and G3 regarding clinical or radiological outcomes. - Median benefit estimate for patients who did not previously suffered a HTO procedure was smaller then benefit for those who did. - A median CER of 1800 € /QALY was found based on the EuroQol scores for G1; 1268 € / QALY for G2, and 1975 € / QALY for G3.	Conservative management for knee OA is neither clinically effective for pain or disease progression nor cost effective, when applied for late stages of OA. TKA proved to be a cost effective procedure in treating knee OA. This study reported the lowest cost per QALY in the literature for TKA. TKA after HTO is technically more difficult and lead to a greater rate of perioperative complications	56

^a^Good quality = ≥75 Moderate quality = 50 to 74 Poor quality = <50

#### TKA versus Unicondylar/Unicompartmental Knee Arthroplasty (UKA)

Five studies compared TKA with UKA to determine the most cost-effective option [21, 25, 27, 33, 34]. The first study [27] looked at cost-effectiveness over 2 years using a prospective observational study and found that TKA was associated with both a higher cost and a QALY gain, but could not be 95% confident that TKA is cost-effective due to the short length of their study. The second study [21] found that TKA was associated with higher costs and longer hospital stays in the short run whilst UKA was associated with significantly poorer long term survival rates and higher possibility for revision surgery or secondary TKA. The study noted that the cost saved by lower implant prices and shorter hospital stays may not be able to cover the cost of extra revision surgeries that are needed in the future [21]. Peersman et al. [34] used a Markov model to assess cost-effectiveness in four different age groups (<55, 55-65, 65-75 and 75+) and found that in all age groups UKA was associated with cost reductions and health gains. The conclusion from this study was that TKA was not cost-effective when compared to UKA. However, this study did not adopt a societal perspective. The study by Soohoo et al. [25] used a decision model to assess cost-effectiveness in patients with end stage unicompartmental knee OA and found UKA to be cost-effective compared to TKA if the durability and function of UKA are assumed to be the same as TKA. The final study [33] compared TKA with UKA and High Tibial Osteotomy (HTO) in younger patients (50-60 years) with unicompartmental OA using a Markov model and from a societal perspective. The study concluded that TKA had just a 31% chance of being cost-effective compared with UKA and THO and that HTO provided value for money in 50 to 60 year old patients. A summary of results are presented in Table 2.

#### TKA versus other treatments

One study compared TKA with a do nothing approach over patients lifetime in the Australian population from a health system perspective [35], and found TKA to be a cost-effective intervention. Another study [20] compared early and delayed TKA (with and without a non operative bridge) in a cohort of 60 year olds from a societal perspective. The study found that TKA without delay is the preferred cost-effective treatment strategy when compared to waiting for TKA without non-operative bridge. This study noted that as it is unlikely that patients waiting for TKA would not receive non-operative treatment, TKA without delay may be an overall cost-saving health care delivery strategy.

Another study [26] compared two types of TKA techniques (standard and innovative TKA) and concluded that innovative implants would be cost-effective if they reduce TKA failure by approximately 50%. Finally, Losina et al [36] used a Markov model to simulate costs and QALY gains for TKA, carried out in low, medium and high volume hospitals; and using no TKA as comparator. Findings relate to the Medicare aged population in the US. TKA was found to be cost-effective across all risk groups and, usurpingly, to be more cost-effective when carried out in high volume hospitals (Table 2).

### Cost-effectiveness of Total Hip Arthroplasty (THA)

#### THA vs Metal on metal hip resurfacing arthroplasty (MoM-HRA)

Metal-on-metal hip resurfacing arthroplasty (MoM-HRA) was considered as an alternative in two studies [29, 37]. The first study used a Markov model to compare the cost-effectiveness of THA and MoM-HRA in patients aged 50 years and over from a health system perspective over a 30 year period and the results show that MoM-HRA is cost-effective in younger patients and although MoM-HRA showed improvements in QALYs, further research is needed to reach a definite conclusion as to which is the most cost-effective intervention [29]. The second study used a Markov model to assess the cost-effectiveness of THA compared with MoM-HRA from a health system perspective over a 15 year period. The study concluded that age wise, MoM-HRA was preferable in younger patients and THA in older patients and gender wise, MoM-HRA was more preferable in men and THA in women [37].

### THA vs other strategies

One study compared THA to a non-operative strategy from a health care perspective and found THA to be a cost-effective intervention [28]. Another study compared the intervention to a do nothing approach over patients’ lifetime in the Australian population from a health system perspective and found THA to be cost-effective [35]. The study by Mota used a Markov modelling approach to compare early and delayed hip arthroplasty in a cohort of patients based on sex and age (50-59 years, 60-74 years and 75 years and over) from a health provider perspective and the results from this study suggest that early THA is a cost-effective option across groups based on age and sex [38].

Four studies compared various techniques for THA. The first study [39] compared two techniques for THA (cementless and hybrid) over patients lifetime using a Markov model and found the cementless technique to be cost-effective compared to the hybrid technique in most cases. However, the cementless technique was found to be dominant in patients less than 43 years whilst the hybrid technique seemed to be dominant in patients aged 83 and over.

The second study assessed the lifetime cost-effectiveness of cemented, cementless and hybrid techniques and found the hybrid to be the most cost-effective compared to cemented and cementless in 60, 70 and 80 year old patients. The results obtained in this study were similar for both men and women [23]. The third study [40] compared five different techniques for THA from an NHS/PSS perspective and concluded that it is difficult to make a choice between techniques based on cost-effectiveness grounds alone. The fourth study assessed the cost-effectiveness of three types of prosthesis for hip replacement (cemented, cementless and hybrid) in adults aged between 55 and 84 years. The study found the hybrid prosthesis to be the most cost-effective across all age groups, with the exception of 80 year old women where the cemented prosthesis was the most cost-effective [41].

### Cost-effectiveness of other surgical interventions

For studies that considered implant systems, one study evaluated the KineSpring implant system and found it to be cost-effective compared to other surgical and conservative treatments [22]. Another study compared arthroscopic surgery with a non-operative treatment and found that arthroscopic surgery was not a cost-effective option [19]. A summary of the results and conclusions of studies assessing the cost-effectiveness of surgical interventions is presented in Table 2.

### Overall quality of included studies

Based on the Quality of Health Economic Studies (QHES) instrument, a total of six studies were categorised as good quality with scores of 75 and above whilst fourteen other studies were categorised as moderate quality with scores between 50 to 74 (Table 2 and Additional file 1). Three studies had scores below 50 [21, 22, 28] and a close examination of these studies revealed striking flaws such as not reporting the source of transition probabilities, perspectives, discount rate, cycle length and time horizon. One study [22] did not perform any sensitivity analysis to deal with the sampling uncertainty or give any details of the source of effectiveness data.

Fourteen out of the eighteen model based studies used Markov/semi-Markov models due to the chronic nature of OA and the ability of such models to handle recurrent events. Other model types that were used include decision tree modelling [25] and discrete-event simulation (DES) which has the flexibility to accommodate a richer structure without making it unmanageable in size [35]. Only one study did not specify which model type was used as the basis of its economic evaluation [22]. The time horizon used in the model based studies varied from one year to lifetime and most of the studies chose a 1-year cycle length for transitions to occur from one health state to another. For those studies that accounted for revision of surgery and failure of implant as one of the outcomes, one year is considered as a reasonable time to decide for further management, should any of the events occur. The most commonly used sensitivity analysis in the model-based economic evaluations in this review is probabilistic sensitivity analysis. This involves specifying distributions for model parameters to represent uncertainty in their estimation followed by employing Monte Carlo simulation to select values at random from those distributions [42]. Other types of sensitivity analysis used were deterministic, one-way and two-way sensitivity analysis, subgroup analysis, regression analysis and scenario analysis.

## Discussion

This systematic review was conducted with the objective of assessing the cost-effectiveness of surgical interventions for the treatment of knee and hip OA. Overall, the review found that most studies considered the most important components pertinent to economic analyses such as perspective, currency, price year and time horizon. Total knee arthroplasty, total hip arthroplasty and metal-on-metal hip resurfacing arthroplasty showed evidence of cost-effectiveness and improvement in quality of life of the patients. However, this depended on the population that was considered and the interventions that they were compared to. For example, TKA and THA were found to be cost-effective for patients with severe or end-stage knee or hip OA across all age groups and when compared to non-operative strategies. Regardless of perspective adopted, TKA and THA remain among the most effective interventions in terms of improvements in quality of life of OA patients. Compared to early primary THA or TKA, the findings suggest that delayed total hip arthroplasty was not cost-effective [38]. MoM-HRA surgery was more cost-effective in younger patients.

More than half of the studies in this review used decision-analytic models and as expected, some of the earlier studies [25, 43] used decision trees to analyse the cost-effectiveness of the interventions of interest, whilst the more recent studies utilised Markov models. This is an indication that adequate information on model structures is now readily available and guidelines are being adhered to. Based on the chronic nature of the disease and potential of recurrence of the events that may occur during disease progression in OA, Markov models have been shown to be the most suitable decision-analytic model for this condition since they provide a far more convenient way of modelling prognosis for clinical problems with ongoing risk whereby events may re-occur and when the utility of an outcome depends on when it occurs [44]. One study employed a discrete-event simulation (DES) model to assess the cost-effectiveness of total hip and knee arthroplasty in Australia [35]. The literature has shown that DES is able to represent the course of a disease more naturally and is best used in a condition where interaction at individual or patient level is a significant component in modelling [45, 46].

### Strengths and limitations of the study

To the best of our knowledge, this is the only systematic review pertaining to the cost-effectiveness of surgical treatment modalities in management of osteoarthritis. In addition, methodological issues related with economic evaluations of surgical interventions in management of OA were also assessed and reported in this study. This study critically assessed the quality of included studies both qualitatively with the Philips and CHEERS checklists as well as quantitatively using the QHES scoring tool.

Because a broader search strategy was initially adopted, a large number of studies were hand searched. This may mean that we have been able to identify a larger number of relevant papers than if the initial search criteria had been limited to surgical interventions for hip and knee OA.

Limitations of the study include the following: Due to the study aims and the need to include as many studies as possible, we did not exclude any studies based on the results of the quality checks. Hence, the robustness of this systematic review may possibly be affected by the poor quality studies included in this report. In addition to this, the quality assessment was carried out by one reviewer. Second, studies that were included in this review were conducted across nine countries which may lead to problems such as generalisability and transferability of the study findings to other settings due to differences in factors such as clinical practice, prices and epidemiology of disease [47, 48]. In addition to this, guidance in many countries does not consider economic data to be transferable to their settings in most cases [49, 50]. As a result, care must be taken when interpreting the results of this study in a particular context.

### Policy implications and recommendations

Clinical guidelines by the National Institute for Health and Care Excellence in the UK recommend a holistic approach to osteoarthritis assessment and management [8]. The guidelines advocate for patient education and self-management to enhance understanding of the condition and its management. Unfortunately, the information on economic analyses in the guidelines is confined to pharmacological and conventional treatments. Cost-effectiveness findings related to surgical interventions were merely focused on time to referral for surgery as being highlighted in the guidelines by National Collaborating Centre for Chronic Conditions [51]. It is therefore recommended that guidelines should also include suggestions for the use of surgical interventions as well as other forms of treatments and management techniques. One of the concerns around surgical interventions such as arthroplasty relates to the cost implications surrounding it. It may be argued that avoiding or delaying these surgical procedures may have a positive impact on health budgets through savings. However, it should be noted that delaying such a procedure may have a detrimental effect on the quality of life of patients and may lead to additional costs down the line. Studies included in this review concluded that delaying arthroplasty (both knee and hip) was not a cost-effective option and that the health related quality of life lost as a result of the delay is greater than the savings in costs that may occur from delaying the procedure [38]. It is therefore suggested that future cost-effectiveness studies assess the cost-effectiveness of delaying surgery in various sub-groups of patients and in various settings in order to reach a consensus about when the operation should be conducted and when delaying surgery would yield the optimum results.

Although interventions such as THA have been shown to be cost-effective, there needs to be additional studies comparing the various types of THA surgeries in order to maximise benefits from the available resources. In addition, other interventions such as MoM-HRA have been shown to be cost-effective in younger patients [37]. Therefore health policy makers should take such evidence into consideration when providing guidance. Policy makers and stakeholders should therefore consider TKA and THA as opposed to non-operative/non surgical strategies particularly for patients with severe/advanced stage OA [18]. From this review, it is clear that there is limited evidence with respect to other forms of surgical treatments. It is therefore suggested that additional studies should be conducted in order to determine the cost-effectiveness of other forms of surgical interventions for both knee and hip OA.

This study found that approximately 36% percent of studies limited their analysis to a healthcare perspective. It should however be noted that a large proportion of the economic burden of OA is related to indirect costs and productivity losses [6]. Thus, the societal perspective has the potential to capture all important impacts on the whole society. Recent guidelines suggest that due to the chronic nature of OA, a broader societal perspective is preferred [9, 52]. It should however be recognised that national guidelines such as those in the UK recommend that economic analysis should be undertaken from a health service perspective [53] and as a result, analysts in countries like the UK might limit their study to the health service perspective. However, it is suggested that all studies should also consider the societal perspective within sensitivity analysis in order to provide a better picture of the true burden associated with OA and also ensure that the results are more generalisable to other settings.

## Conclusions

This review assessed the cost-effectiveness of surgical interventions for the management of knee and hip osteoarthritis and the results suggest that TKA and THA are cost-effective interventions particularly when compared to non-operative strategies and also when the operation is not delayed. However, there is the need for more studies assessing the cost-effectiveness of other surgical modalities. In addition, most of the identified studies were considered to have moderate quality. It is therefore recommended that more cost-effectiveness studies with high methodological standards are conducted.

## Additional file

Additional file 1: Search strategy for electronic databases. (DOCX 33 kb)

## Abbreviations

CHEERS: Consolidated health economic evaluating reporting standard
DES: Discrete event simulation
EQ-5D: EuroQoL five dimension
HAQ: Health assessment questionnaire
HR-QoL: Health related quality of life
HTO: High tibial osteotomy
HUI: Health utility index
ICER: Incremental cost-effectiveness ratio
MoM-HRA: Metal on metal hip resurfacing arthroplasty
OA: Osteoarthritis
QALY: Quality adjusted life years
QHES: Quality of health economics studies
SF-36: Short form 36
SF-6D: Short form 6D
THA: Total hip arthroplasty
TKA: Total knee arthroplasty
UKA: Unicondylar knee arthroplasty
