# Supplementary material for: Cost-effectiveness of surgical interventions for the management of osteoarthritis: a systematic review of the literature
Source: BMC Musculoskelet Disord. 2017 May 10;18:183. doi: 10.1186/s12891-017-1540-2 (PMC5424321; doi:10.1186/s12891-017-1540-2)
Supplement: Additional file 1: — Search strategy for electronic databases. (DOCX 33 kb) [file 12891_2017_1540_MOESM1_ESM.docx]

***Additional file 1 : Search strategy for electronic databases***

**Database: Ovid MEDLINE(R) In-Process & Other Non-Indexed Citations and Ovid MEDLINE(R) <1950 to 3rd May 2016>**

1. Osteoarthritis/

2. osteoarthritis.tw.

3. degenerative arthritis.tw.

4. 1 or 2 or 3 *(This is a combination of search terms 1, 2 and 3 above. It broadens the search to include articles containing each term separately, as well as all terms together)*

5. exp Acetaminophen/

6. acetaminophen.tw.

7. paracetamol.tw.

8. 5 or 6 or 7 *(This is a combination of search terms 5, 6 and 7 above. It broadens the search to include articles containing each term separately, as well as all terms together)*

9. exp Tramadol/

10. tramadol.tw.

11. tramadol hydrochloride.tw.

12. 9 or 10 or 11 *(This is a combination of search terms 9, 10 and 11 above. It broadens the search to include articles containing each term separately, as well as all terms together)*

13. exp Cyclooxygenase 2 Inhibitors/

14. Cyclooxygenase 2 Inhibitor$.tw.

15. COX-2 inhibitor$.tw.

16. 13 or 14 or 15 *(This is a combination of search terms 13, 14 and 15 above. It broadens the search to include articles containing each term separately, as well as both terms together)*

17. exp non-steroidal anti-inflammatory agents/

18. non-steroidal anti-inflammatory agent$.tw.

19. NSAID$.tw.

20. 17 or 18 or 19 *(This is a combination of search terms 17, 18 and 19 above. It broadens the search to include articles containing each term separately, as well as both terms together)*

21. diacerein.tw.

22. exp Glucosamine/

23. glucosamine.tw.

24. (glucosamine adj1 (sulphate or hydrochloride)).tw.

25. 21 or 22 or 23 or 24 *(This is a combination of search terms 21, 22, 23 and 24)*

26. exp Chondroitin/

27. chondroitin.tw.

28. chondroitin sulphate.tw.

29. 26 or 27 or 28 *(This is a combination of search terms 26, 27 and 28)*

30. corticosteroid.mp.

31. glucocorticoid$.tw.

32. intraarticular corticosteroid.tw.

33. 30 or 31 or 32 *(This is a combination of search terms 30, 31 and 32)*

34. exp Platelet-Rich Plasma/

35. platelet rich plasma.tw.

36. 34 or 35 *(This is a combination of search terms 34 and 35)*

37. exp Viscosupplementation/

38. viscosupplementation$.tw.

39. exp Hyaluronic Acid/

40. hyaluronic acid.tw.

41. (sodium adj1 hyaluronic).tw.

42. 37 or 38 or 39 or 40 or 41*(This is a combination of search terms 37 to 41)*

43. methylsalicylate.mp.

44. topical NSAID$.tw.

45. exp Capsaicin/

46. capsaicin.tw.

47. 43 or 44 or 45 or 46 *(This is a combination of search terms 43 to 46)*

48. 8 or 12 or 16 or 20 or 25 or 29 or 33 or 36 or 42 or 47 *(This search expands all the previous combinations of search terms for the various pharmacological interventions)*

49. exp Arthroplasty/

50. joint arthroplasty.tw.

51. arthroplast$.tw.

52. 49 or 50 or 51 *(This is a combination of search terms 49, 50 and 51)*

53. exp Osteotomy/

54. tibial osteotomy.tw.

55. 53 or 54 *(This is a combination of search terms 53 and 54)*

56. exp Spinal Fusion/

57. spinal fusion.tw.

58. exp Arthrodesis/

59. arthrodes?s.tw.

60. spinal arthrodes?s.tw.

61. 56 or 57 or 58 or 59 or 60 *(This is a combination of search terms 56 to 60)*

62. 52 or 55 or 61 *(This search expands all the previous combinations of search terms for the various surgical interventions)*

63. exp Physical Therapy Modalities/

64. physiotherap$.tw.

65. exp Exercise Therapy/

66. exercise therap$.tw.

67. 63 or 64 or 65 or 66 *(This is a combination of search terms 63 to 66)*

68. exp Occupational Therapy/

69. occupational therap$.tw.

70. 68 or 69 *(This is a combination of search terms 68 and 69)*

71. exp Orthotic Devices/

72. orthos?s.tw.

73. assistive device$.tw.

74. joint protection$.tw.

75. exp Braces/

76. joint brace$.tw.

77. 71 or 72 or 73 or 74 or 75 or 76 *(This is a combination of search terms 71 to 76)*

78. exp Complementary Therapies/

79. alternative therap$.tw.

80. herbal therap$.tw.

81. exp Herbal Medicine/

82. exp Acupuncture/

83. acupuncture.tw.

84. 78 or 79 or 80 or 81 or 82 or 83 *(This is a combination of search terms 78 to 83)*

85. 67 or 70 or 77 or 84 *(This search expands all the previous combinations of search terms for the various non-surgical interventions)*

86. 48 or 62 or 85 *(This search expands the various combinations for pharmacological, surgical and non-pharmacological interventions)*

87. 4 and 86 *(This is a combination of search 4 {Which captures articles that are concerned with osteoarthritis} and 86 {Which captures articles that have considered pharmacological, non-pharmacological and surgical interventions}. This narrows the search to include articles that have included both terms only)*

88. exp Cost-Benefit Analysis/

89. economic evaluation$.tw.

90. cost effectiveness.tw.

91. cost benefit analysis.tw.

92. cost utility analysis.tw.

93. cost consequence analysis.tw.

94. 88 or 89 or 90 or 91 or 92 or 93 *(This is a combination of search 88, 89, 90, 91, 92 and 93 above. It broadens the search to include articles containing each term separately, as well as both terms together)*

95. 87 and 94 *(This is a combination of search 87 {Which captures articles that have considered interventions for osteoarthritis} and 94 {Which captures articles that have conducted an economic evaluation/cost-effectiveness analysis}. This narrows the search to include articles that have included both terms only)*

96. limit 95 to "humans"

***Additional file 1 : CHEERS Checklist***

| **Section / Item** | **Item No.** | **Recommendation** | **Reported on page no. / line no.** |
| --- | --- | --- | --- |
| **Title and abstract** |  |  |  |
| Title | 1 | Identify the study as an economic evaluation or use more specific terms such as "cost-effectiveness analysis", & describe the interventions compared. | **_______________** |
| Abstract | 2 | Provide a structured summary of objectives, perspective, setting, methods (including study design & inputs), results (including base case and uncertainty analyses), & conclusion. | **_______________** |
|  |  |  |  |
| **Introduction** |  |  |  |
| Background & objectives | 3 | Provide an explicit statement of the broader context for the study.  Present the study question and its relevance for health policy or practice decisions. | **_______________** |
|  |  |  |  |
| **Methods** |  |  |  |
| Target population & subgroups | 4 | Describe characteristics of the base case population and subgroup analysed, including why they were chosen. | **_______________** |
| Setting & location | 5 | State relevant aspects of the system(s) in which the decision(s) need(s) to be made. | **_______________** |
| Study perspective | 6 | Describe the perspective of the study & relate this to the costs being evaluated. | **_______________** |
| Comparators | 7 | Describe the interventions or strategies being compared & state why they were chosen. | **_______________** |
| Time horizon | 8 | State the time horizon(s) over which costs and consequences are being evaluated and say why appropriate. | **_______________** |
| Discount rate | 9 | Report the choice of discount rate(s) used for costs & outcomes and say why appropriate. | **_______________** |
| Choice of health outcomes | 10 | Describe what outcomes were used as the measure(s) o benefit in the evaluation & their relevance for the type of analysis performed. | **_______________** |
| Measurement of effectiveness | 11a | *Single study-based estimates*: Describe fully the design features of the single effectiveness study & why the single study was a sufficient source of clinical effectiveness data. | **_______________** |
|  | 11b | *Synthesis-based estimates:* Describe fully the methods used for identification of included studies & synthesis of clinical effectiveness data. | **_______________** |
| Measurement & valuation of preference based outcomes | 12 | If applicable, describe the population & methods used to elicit preferences for outcomes. | **_______________** |
| Estimating resources & costs | 13a | *Single study-based economic evaluation*: Describe approaches used to estimate resource use associated with the alternative interventions.  Describe primary or secondary research methods in valuing each resource item in terms of unit cost.  Describe any adjustments made to approximate to opportunity costs. | **_______________** |
|  | 13b | *Model-based economic evaluation:*  Describe approaches & data sources used to estimate resource use associated with model health states.  Describe primary or secondary research methods in valuing each resource item in terms of unit cost.  Describe any adjustments made to approximate to opportunity costs. | **_______________** |
| Currency, price date & conversion | 14 | Report the dates of the estimated resource quantities and unit costs. Describe methods for adjusting estimated unit costs to the year of reported costs if necessary. Describe methods for converting costs into a common currency base and the exchange rate. | **_______________** |
| Choice of model | 15 | Describe & give reasons for the specific type of decision-analytic model used. Providing a figure to show model structure is strongly recommended. | **_______________** |
| Assumptions | 16 | Describe all structural or other assumptions underpinning the decision-analytical model. | **_______________** |
| Analytical methods | 17 | Describe all analytical methods supporting the evaluation. This could include methods for dealing with skewed, missing or censored data; extrapolation methods; methods for pooling data; approaches to validate or make adjustments (such as half cycle corrections) to a model; & methods for handling population heterogeneity and uncertainty. | **_______________** |
|  |  |  |  |
| **Results** |  |  |  |
| Study parameters | 18 | Report the values, ranges, references, &, if used, probability distributions for all parameters. Report reasons or sources for distributions used to represent uncertainty where appropriate. Providing a table to show the input values is strongly recommended. | **_______________** |
| Incremental costs and outcomes | 19 | For each intervention, report mean values for the ma categories of estimated costs and outcomes of interest, as well as mean differences between the comparator groups. If applicable, report incremental cost-effectiveness ratios. | **_______________** |
| Characterising uncertainty | 20a | Single study-based economic evaluation: Describe the effects of sampling uncertainty for the estimated incremental cost & incremental effectiveness parameters, together with the impact of methodological assumptions (such as discount rate, study perspective). | **_______________** |
|  | 20b | Model-based economic evaluation: Describe the effects on the results of uncertainty for all input parameters, and uncertainty related to the structure of the model and assumptions. | **_______________** |
| Characterising heterogeneity | 21 | If applicable, report differences in costs, outcomes, or cost-effectiveness that can be explained by variations between subgroups of patients with different baseline characteristics or other observed variability in effects that are not reducible by information. | **_______________** |
|  |  |  |  |
| **Discussion** |  |  |  |
| Study findings, limitations, generalisability & current knowledge | 22 | Summarise key findings and describe how they support the conclusions reached. Discuss limitations & the generalisability of the findings & how the findings fit with current knowledge. | **_______________** |
|  |  |  |  |
| **Others** |  |  |  |
| Source of funding | 23 | Describe how the study was funded & the role of the funder in the identification, design, conduct & reporting of the analysis. Describe other non-monetary sources of support. | **_______________** |
| Conflicts of interest | 24 | Describe any potential for conflict of interest of study contributors in accordance with journal policy. In the absence of a journal policy, we recommend authors comply with International Committee of Medical Journal Editors recommendations. | **_______________** |

**Source** : Husereau, D., Drummond, M., Petrou, S. et al. Consolidated health economic evaluation reporting standards (CHEERS) - Explanation and elaboration: A report of the ISPOR health economic evaluations publication guidelines good reporting practices task force. Value Health, 2013;16:231-5.

***Additional file 1: Philips checklist***

| **No.** | **Question / Component** | **Y / N / UC / NA** |
| --- | --- | --- |
| **STRUCTURE** | | |
| 1. | Is there a clear statement of the decision problem? |  |
| 2. | Is the objective of the model specified & consistent with the stated decision problem? |  |
| 3. | Is the primary decision maker specified? |  |
| 4. | Is the perspective of the model stated clearly? |  |
| 5. | Are the model inputs consistent with the stated perspective? |  |
| 6. | Is the structure of the model consistent with a coherent theory of the health condition under evaluation? |  |
| 7. | Are the sources of the data used to develop the structure of the model specified? |  |
| 8. | Are the structural assumptions reasonable given the overall objective, perspective and scope of the model? |  |
| 9. | Is there a clear definition of the options under evaluation? |  |
| 10. | Have all feasible and practical options been evaluated? |  |
| 11. | Is there justification for the exclusion of feasible options? |  |
| 12. | Is the chosen model type appropriate given the decision problem and specified causal relationships within the model? |  |
| 13. | Is the time horizon of the model sufficient to reflect all important differences between the options? |  |
| 14. | Do the disease states (state transition model) or the pathways (decision tree model) reflect the underlying biological process of the disease in question and the impact of intervention? |  |
| 15. | Is the cycle length defined and justified in terms of the natural history of disease? |  |
| **DATA** | | |
| 16. | Are the data identification methods transparent and appropriate given the objectives of the model? |  |
| 17. | Where choices have been made between data sources, are these justified appropriately? |  |
| 18. | Where expert opinion has been used, are the methods described and justified? |  |
| 19. | Is the choice of baseline data described and justified? |  |
| 20. | Are transition probabilities calculated appropriately? |  |
| 21. | Has a half-cycle correction been applied to both costs and outcomes? |  |
| 22. | If not, has the omission been justified? |  |
| 23. | Have the methods and assumptions used to extrapolate short-term results to final outcomes been documented and justified? |  |
| 24. | Are the costs incorporated into the model justified? |  |
| 25. | Has the source for all costs been described? |  |
| 26. | Have discount rates been described and justified given the target decision maker? |  |
| 27. | Are the utilities incorporated into the model appropriate? |  |
| 28. | Is the source of utility weights referenced? |  |
| 29. | If data have been incorporated as distributions, has the choice of distributions for each parameter been described and justified? |  |
| 30. | If data are incorporated as point estimates, are the ranges used for sensitivity analysis stated clearly and justified? |  |
| 31. | Has heterogeneity been dealt with by running the model separately for different sub-groups? |  |
| **VALIDITY** | | |
| 32. | Have the results been compared with those of previous models and any differences in results explained? |  |
| 33. | Are the conclusions presented compatible with the results? Is it valid and justified? |  |

*** Y = Yes , N = No , UC = Unclear , NA = Not applicable**

***Additional file 1: Breakdown of QHES scores***

|  | Was the study objective presented in a clear, specific and measurable way | Were the perspective of the analysis (societal, third-party payer, etc.) and reasons for its selection stated? | Were variable estimates used in the analysis from the best available source (RCT = best, expert opinion = worst)? | If estimates came from a subgroup analysis, were the groups pre-specified at the beginning of the study? | Was uncertainty handled by (1) statistical analysis to address random events, (2) sensitivity analysis to cover a range of assumptions? | Was incremental analysis performed between alternatives for resources and costs? | Was the methodology for data extraction (including the value of health states and other benefits) stated? | Did the analytic horizon allow time for all relevant and important outcomes? Were benefits and costs that went beyond 1 year discounted (3% - 5%) and justification given for the discount rate? | Was the measurement of costs appropriate and the methodology for the estimation of quantities and unit costs clearly described? | Was the primary outcomes measure(s) for the economic evaluation clearly stated and were the major short-term, long-term and negative outcomes included? | Were the health outcomes measures/scales valid and reliable? If previously tested valid and reliable measures were not available, was justification given for the measures/scales used? | Were the economic model (including structure), study methods and analysis, and the components of the numerator and denominator displayed in a clear, transparent manner? | Were the choice of economic model, main assumptions, and limitations of the study stated and justified? | Did the author(s) explicitly discuss the direction and magnitude of potential biases? | Were the conclusions/recommendations of the study justified and based on the study results? | Was there a statement disclosing the source of funding for the study? | Total score |
| --- | --- | --- | --- | --- | --- | --- | --- | --- | --- | --- | --- | --- | --- | --- | --- | --- | --- |
| Points available | 7 | 4 | 8 | 1 | 9 | 6 | 5 | 7 | 8 | 6 | 7 | 8 | 7 | 6 | 8 | 3 | 100 |
| Bedair et al, 2014 [18] | 5 | 4 | 4 | 0 | 7 | 4 | 5 | 6 | 6 | 4 | 5 | 6 | 6 | 4 | 5 | 3 | 74 |
| Bozic et al, 2010 [29] | 7 | 2 | 6 | 1 | 9 | 6 | 3 | 6 | 7 | 4 | 6 | 5 | 4 | 5 | 8 | 3 | 82 |
| Di Tanna et al, 2011 [39] | 7 | 2 | 4 | 0 | 5 | 4 | 3 | 5 | 4 | 3 | 3 | 5 | 5 | 5 | 4 | 3 | 62 |
| Heintzbergen et al, 2013 [37] | 7 | 2 | 6 | 1 | 8 | 3 | 3 | 5 | 8 | 4 | 6 | 7 | 7 | 5 | 6 | 3 | 81 |
| Higashi et al, 2011 [35] | 5 | 4 | 5 | 0 | 6 | 5 | 3 | 6 | 6 | 4 | 4 | 5 | 5 | 3 | 6 | 3 | 70 |
| Koskinen et al, 2008 [21] | 3 | 0 | 3 | 0 | 4 | 2 | 2 | 0 | 4 | 3 | 3 | 0 | 2 | 3 | 4 | 0 | 33 |
| Li et al, 2013 [22] | 4 | 0 | 3 | 0 | 3 | 4 | 2 | 3 | 4 | 3 | 5 | 2 | 3 | 2 | 4 | 2 | 44 |
| Losina et al, 2009 [36] | 6 | 2 | 4 | 1 | 6 | 5 | 3 | 5 | 5 | 4 | 5 | 6 | 6 | 5 | 7 | 3 | 73 |
| Mota, 2013 [38] | 6 | 4 | 7 | 1 | 7 | 5 | 4 | 6 | 7 | 5 | 5 | 7 | 6 | 5 | 7 | 0 | 82 |
| Pennington et al, 2013 [41] | 6 | 4 | 5 | 1 | 7 | 5 | 4 | 6 | 7 | 5 | 6 | 5 | 6 | 5 | 7 | 3 | 82 |
| Räsänen et al, 2007 [28] | 4 | 2 | 4 | 0 | 4 | 0 | 3 | 3 | 4 | 3 | 4 | 4 | 4 | 3 | 4 | 3 | 49 |
| Ruiz et al, 2013 [31] | 6 | 4 | 5 | 0 | 5 | 4 | 3 | 5 | 6 | 4 | 5 | 6 | 5 | 4 | 6 | 3 | 71 |
| SooHoo et al, 2004 [25] | 5 | 2 | 4 | 0 | 5 | 3 | 3 | 4 | 6 | 3 | 3 | 5 | 4 | 4 | 5 | 3 | 59 |
| Suter et al, 2013 [26] | 5 | 2 | 6 | 1 | 5 | 3 | 4 | 5 | 4 | 4 | 5 | 5 | 5 | 4 | 5 | 2 | 65 |
| Waimann et al, 2014 [32] | 5 | 2 | 5 | 0 | 6 | 4 | 4 | 3 | 5 | 2 | 4 | 5 | 4 | 4 | 4 | 3 | 60 |
| Xie et al, 2010 [27] | 5 | 4 | 4 | 0 | 6 | 4 | 3 | 3 | 5 | 4 | 5 | 5 | 4 | 4 | 6 | 0 | 60 |
| Konopka et al, 2015 [33] | 6 | 2 | 6 | 1 | 7 | 5 | 4 | 5 | 6 | 6 | 5 | 6 | 6 | 5 | 5 | 3 | 78 |
| Marsh et al, 2016 [19] | 5 | 4 | 7 | 0 | 6 | 4 | 4 | 4 | 6 | 4 | 5 | 6 | 5 | 4 | 7 | 3 | 74 |
| Mather et al, 2014 [20] | 6 | 3 | 6 | 0 | 7 | 4 | 4 | 5 | 7 | 4 | 5 | 6 | 5 | 5 | 6 | 3 | 76 |
| Peersman et al, 2014 [34] | 5 | 2 | 6 | 0 | 6 | 5 | 4 | 5 | 6 | 4 | 6 | 6 | 5 | 4 | 5 | 3 | 72 |
| Pennington et al, 2015 [23] | 4 | 0 | 5 | 0 | 5 | 1 | 3 | 4 | 5 | 4 | 5 | 6 | 5 | 4 | 6 | 0 | 57 |
| Pulikottil-Jacob et al, 2015 [40] | 4 | 3 | 6 | 0 | 5 | 1 | 3 | 4 | 5 | 4 | 5 | 5 | 5 | 4 | 5 | 3 | 62 |
| Stan et al, 2015 [24] | 4 | 0 | 4 | 0 | 4 | 1 | 3 | 4 | 5 | 4 | 4 | 5 | 5 | 4 | 6 | 3 | 56 |
